# Practice and Evaluation of a Protocol for Collating Information Related to the Emergency Clinical Pathway for Patients With Acute ST-Segment Elevation Myocardial Infarction

**DOI:** 10.3389/fcvm.2022.840715

**Published:** 2022-04-27

**Authors:** Jun Cao, Zi Ge, Hui Zhao, Ke Ma, Zhijie Xia

**Affiliations:** Department of Emergency and Critical Care Medicine, Huashan Hospital North, Fudan University, Shanghai, China

**Keywords:** ST elevation myocardial infarction, clinical pathway, quality control, time to treatment, information system

## Abstract

Acute ST elevation myocardial infarction (STEMI) is a common acute and critical disease that requires rapid treatment within a limited window of time. In this study, we attempt to introduce a clinical pathway for the whole-process management of emergency STEMI based on the creation of a specific information system that matches the characteristics of emergency clinical work and evaluates their clinical value by quality control analysis. We deployed this system for 3 years and found that complications, heart failure, and medical costs during hospitalization were significantly reduced (*p* = 0.019) in patients with STEMI. By analyzing each link in the clinical pathway, our research indicates the clear clinical importance of developing methods to continuously improve data quality. Collectively, out research led to the optimization of an information system that will facilitate the clinical management of patients with STEMI.

## Introduction

Acute ST-segment elevation myocardial infarction (STEMI) is one of the most critical diseases that requires emergency quality control. The area affected by myocardial necrosis is known to be linearly correlated with the duration of time elapsed during myocardial infarction (1). Delaying reperfusion of the coronary artery is known to increase the incidence of heart failure and death (2, 3). Mortality of STMEI is 4–5% (4). Therefore, it is critical that we diagnose STEMI as early as possible as there is a very high requirement for timely therapeutic intervention. In China, we have some standards to evaluate the effectiveness of treatment and management. Some diseases such as STEMI and stroke are under control. We send these data each year to health superior. In order to improve the efficiency of emergency management, reduce clinical errors, standardize clinical management strategies, and ensure the safety of patients, we investigated the “single disease management” of hospitalized patients and proposed the concept of a “Clinical Pathway for STEMI in the Emergency Department” based on the clinical characteristics of patients in the emergency room. In January 2018, we constructed an emergency clinical pathway information system to promote the continuous improvement of diagnosis and therapy. In the present study, we evaluated the implementation of our emergency clinical pathway and the practical a value of the system by following-up and analyzing the clinical data from a cohort of emergency STEMI patients between January 2015 and December 2020.

## Materials and Methods

### Study Design

This was a retrospective cohort study. The diagnostic criteria used for STEMI was in accordance with the global definition of myocardial infarction (4–7). We screened all emergency patients who had been diagnosed with STEMI between January 2015 and December 2020. The diagnostic criteria of STEMI is severe squeezing pain in the retrosternal or precordial area (usually more than 10–20 min), which can radiate to the left upper arm, mandible, neck, back, or shoulder; often accompanied by nausea, vomiting, sweating and dyspnea. Some patients may have syncope, and nitroglycerin cannot completely relieve the symptom. Electrocardiography (ECG) will show ST-segment arch elevation (monophasic curve) with or without pathological Q wave and depressed R wave, often accompanied by mirror ST segment depression in corresponding leads. If the first ECG could not help diagnosis, it should be reexamined in 15–30 min. The follow-up time nodes were calculated based on the confirmed ECG. Patients with thoracic pain caused by non-coronary artery disease (CAD) and non-ST-segment elevation myocardial infarction (NSTEMI) were excluded. The primary endpoint of our evaluation was death, the discontinuation of treatment, or the reperfusion of vessel, including thrombolysis and direct percutaneous coronary intervention (PCI). All enrolled patients were divided into Group 1 (prior to the implementation of the emergency clinical pathway; 2015–2017) and Group 2 (after the implementation of the emergency clinical pathway; 2018–2020).

### Construction of an Emergency Clinical Pathway Information System

**FIGURE 1:**
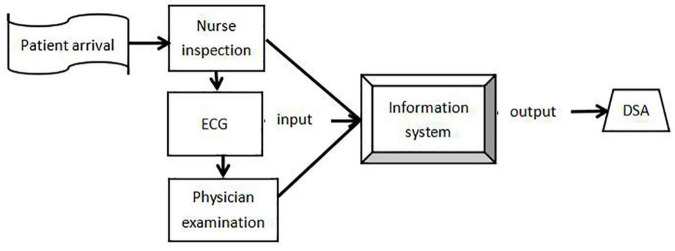
Flowchart depicting the management of STEMI patients in the emergency clinical pathway. (DSA-Digital Subtraction Angiography; ECG-Electrocardiogram).

**FIGURE 2:**
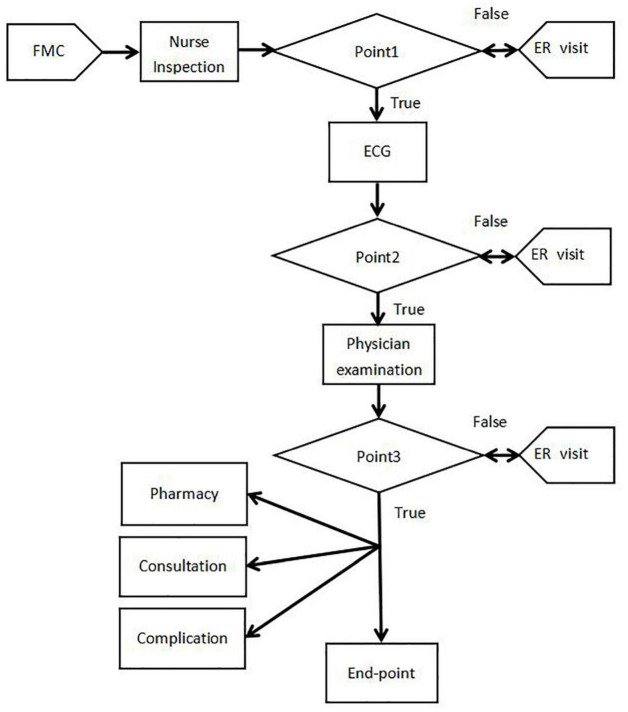
Developmental plan for the information system for patients with STEMI. (FMC-first medical contact; ER-emergency room).

We developed an information system for the STEMI clinical pathway so that we could grade the management of emergency patients, improve disease assessments, and guide clinical physicians to standardize therapy. First, we separated the entire clinical sequence into several different components and generated a flowchart (Figure 1). Then, we focused on three key components within the system to become the focal area for quality control (Figure 2), including the time of first grading, electrocardiogram (ECG) completion, and door-to-balloon (DTB). The function of this information system was to facilitate the collection of first-hand clinical data and analysis during the course of management. This system was designed in a specific format and linked to a standard database which provided up-to-date clinical guidelines for the treatment and management of STEMI.

### Data and Analysis

To demonstrate the continuity of quality control, we collected a range of emergency data over a 6 year period, baseline demographics (gender, age, previous medical history), the time of symptom onset, the time to arrive at the emergency room, the time of the first ECG and PCI, the length of stay in the hospital, cost, and the incidence of complications or mortality. The complications are pathologic processes that affect patients after STEMI and require medical interventions. They may be related to the disease or the treatment. Complications included heart failure, arrhythmia, dysfunction in other organs (infection of the respiratory system, gastrointestinal bleeding, renal failure, and cerebral hemorrhage) and adverse drug reactions during STEMI treatment. Heart failure could be recognized by the clinical symptoms and signs such as shock, shortness of breath, rales in ausculation after STEMI. All these were related to pump failure and acute cardiogenic pulmonary edema. In statistics, continuous variables are expressed as mean with standard deviation (SD) and categorical data are expressed as percentages. The independent Student’s *t*-test or Mann-Whitney *U*-test was used to compare continuous variables when appropriate, and the Chi-squared test or Fisher’s exact test was used to compare categorical variables between two groups. Statistical tests were two-sided and *p* < 0.05 was considered statistically significant. All statistical analysis was conducted using SPSS 24.0 (IBM SPSS statistics, United States).

## Results

**FIGURE 3:**
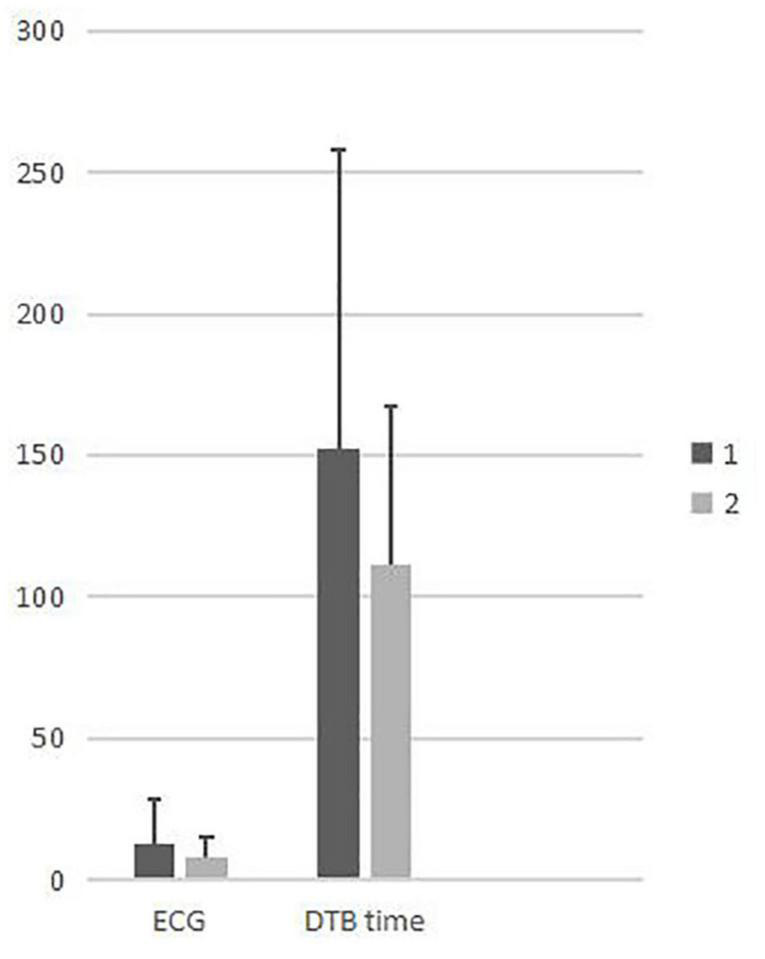
A comparison of time points between the groups. (The vertical axis is in minutes).

**FIGURE 4:**
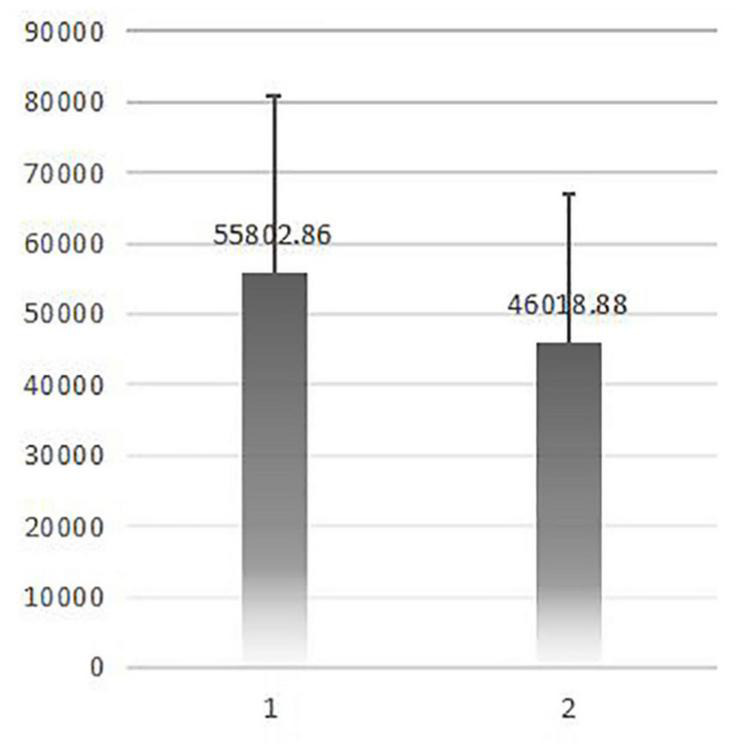
A comparison of cost between the groups. (The vertical axis is in RMB ¥).

Table 1 shows the total number of emergency visits, STEMI patients, and patients who received PCI between 2015 and 2020. A total of 243 patients were included in this study. Table 2 shows a comparison of the demographic characteristics between the two groups. There were no significant differences between the two groups in terms of age, gender, and basic diseases, except for the history of CAD (1.6% vs. 8.5%, *p* < 0.05). However, time to visit emergency was different significantly between two groups while the time in group 2 was shorter obviously (*p* = 0.024). There was no significant difference between the two groups in terms of ECG completion time. The mean DTB times for group 1 and group 2 were 152.3 ± 106.1 min and 111.1 ± 56.1 min, respectively; these were significantly different (*p* < 0.05) (Table 3). The incidence of complications and heart failure events were significantly reduced in the STEMI patients in group 2, although there was no significant difference between the two groups in terms of mortality and length during hospitalization (Table 4). We also found that delayed DBT time was positively correlated with medical costs during hospitalization (Figures 3, 4). Next, we analyzed the reasons for a delay in door-to-balloon time and identified three main factors: (1) patients were hesitant with regards to receiving PCI treatment (22.7%), (2) critical illness with complications requiring initial cardiopulmonary resuscitation (63.6%), and (3) occupation of the PCI room and the availability of the PCI team (13.6%).

**TABLE 1:** Total number of emergency visits and STEMI patients in 2016–2020.

Year	2015	2016	2017	2018	2019	2020
Emergency visits	107,234	126,024	126,070	124,969	131,429	110,501
STEMI patients	34	47	44	44	29	45
STEMI‰	0.32	0.37	0.35	0.35	0.22	0.41
Direct PCI	19	43	38	43	29	45
PCI%	55.9	91.5	86.4	97.7	100	100

**TABLE 2:** Comparison of age, gender and history between two groups.

Group		1	2	*p*
Age (year)		62.93 ± 12.782	63.30 ± 11.302	0.817
Time to visit (h)		12.802 ± 21.166	7.381 ± 12.364	0.024
Gender	Male	102 (81.6%)	101 (85.6%)	0.404
	Female	23 (18.4%)	17 (14.4%)	
History	Hypertension	56 (44.8%)	66 (55.9%)	0.083
	Diabetes type 2	24 (19.2%)	25 (21.2%)	0.700
	CAD	2 (1.6%)	10 (8.5%)	0.013
	Cerebral infarction	9 (7.2%)	8 (6.8%)	0.898
	Hyperlipemia	2 (1.6%)	3 (2.5%)	0.605
	Tumor	3 (2.4%)	5 (4.2%)	0.422

**TABLE 3:** Comparison of two time points between two groups.

Group		1	2	*t*	*p*
Time point (min)	ECG completion (min)	12.6 ± 16.0	8.2 ± 7.1	1.679	0.100
	Door-to-balloon time (min)	152.3 ± 106.1	111.1 ± 56.1	3.475	0.001

**TABLE 4:** Comparison of outcome between two groups.

Group		1	2	*p*
Mortality		4 (3.2%)	7 (5.9%)	0.306
Survivor	Complication	38 (31.4%)	20 (18.0%)	0.019
	Heart failure	20 (16.5%)	6 (5.4%)	0.007
Length in hospital (d)		11.0 ± 5.9	10.1 ± 5.8	0.283
Cost (¥)		55802.9 ± 25206.6	46018.9 ± 20862.8	0.002

## Discussion

Our analysis showed that the morbidity of patients with STEMI remained stable over 6 years, although the total number of visits to the emergency room in 2020 was lower than that in previous years, presumably due to the COVID-19 pandemic. The proportion of cases involving PCI increased year by year. Two of the most important time points in the pathway, ECG and DTB, showed improvement after our emergency information system was deployed. Although, there was no significant difference with regards to ECG time, this change achieved our goal for quality control. Because the total number of deaths remained low in both groups, we compared survival data to identify potential advantages of this information system with regards to complications, hospital admission duration, and medical costs. The new information system also helped to reduce the incidence of complication and events associated with heart failure by achieving better temporal control. Furthermore, the new system also helped us to control hospitalization expenses.

The diagnosis and treatment of STEMI patients is subject to specific published guidelines (4–7). The core guidelines for STEMI therapy are relatively fixed, although the implementation of any diagnosis and treatment is not a simple medical decision, especially for emergency clinicians in a complex and changing environment. In addition to the objective state of illness, medical decision making also involves the cognitive ability, economic endurance, emergency response, professional judgment, and the ability of coordinated departments to respond rapidly, including the triage system, medical laboratory, imaging facility, and cardiovascular specialty; this pathway also involves non-medical personnel who facilitate the transfer of information). The behavior of patients from different sources (urban and rural locations) and different disease histories will also affect the follow-up treatment (8). Medical staff from different backgrounds may also make different decisions when dealing with the same patient. Medical behavior affects prognosis, and errors in medical behavior can be an important factor that leads to death. Previous researchers have made several attempts to reduce clinical errors and optimize the management of STEMI patients (9–18). These efforts have included value judgment from a single indicator such as troponin (19), the improvement of a single factor (such as deciding who initiates the PCI response) (20), simulation of the treatment process for a single case (21), evaluation of a chest pain center (22), and improvements in the health system (23). In order to change the prognosis of patients and promote human health, it is not only necessary to find new techniques and new ideas in clinic, it is also vital that we carry out reasonable actions at the appropriate times (24).

The introduction of a clinical pathway would reduce the subjective influence of medical personnel, avoid errors, improve the efficiency of diagnosis and treatment, and reduce the cost of operation (25). In 2009, the Ministry of Health in China promoted the management of “Single disease species” with each disease being associated with a matching clinical path. However, researchers have yet to consider the management of time-effective requirements for inpatients with STEMI. Therefore, in this study, we created an emergency clinical pathway to improve the management of patients with STEMI. In order to promote the use of this emergency clinical pathway, improve the enthusiasm of medical staff, and reduce workload, we constructed an information system that could be operated at the bedside. The format and logic of our emergency clinical pathway were specifically designed to improve the efficiency and accuracy of information flow.

The emergency clinical pathway for STEMI emphasizes the control of time nodes (26) and highlights the requirements for specific quality control points, including the time of completion for the first ECG, the time of anti-thrombotic treatment, the time of consultation, and the door-to-balloon time. Particular emphasis was placed on the timing of ECG and DTB for PCI treatment. Data showed that our information system reduced these critical times and reduced the incidence of complications and the costs involved. Repetition of the diagnostic and treatment process will lead to more standardized care and help to educate patients, so that decisions can be made rapidly and effectively.

The value of a clinical pathway is not only to ensure that we can meet the “90 min of DTB time” goal (4), but also to analyze the factors that might influence each link, particularly in cases where the goal is not achieved, thus ensuring the continuous promotion of quality control. Clinical pathway research aims to provide a comprehensive analysis of various behaviors involved in diagnosis and treatment (27). The results of this study showed that emergency activities, such as ECG and antithrombotic treatment, can be completed within the required time frame, and that the main factors responsible for a delay in the door-to-balloon time were patient hesitancy, a lack of knowledge with regards to the severity of the disease, fear of surgery, or limited economic conditions. It is important that we highlight that our hospital is located in the suburbs. With the promotion of municipal reconstruction projects, a large proportion of the population are introduced to our district every year, including those from urban populations and other provinces. There are highly significant differences between these populations in terms of culture, this may affect the time required for pre-operative conversation. In view of this, our hospital has undertaken various forms of health publicity activities, emphasizing the urgency for critical cardiovascular disease in the emergency department and the need to shorten the time before surgery as much as possible. In this study, we found that the interval from symptom onset to the ER had been reduced and also that the proportion of PCI procedures had increased significantly. This reflects the increased understanding and vigilance of STEMI patients in our region. This improvement of great significance to treatment efficacy and the reduction of complications.

In order to ensure that PCI is performed in good time, our clinical pathway information system showed that ECG time and DTB time both need to be improved. Via continuous data analysis and consideration of the actual situation in our hospital, the entire process of diagnosis and treatment was improved in a three-dimensional manner by five mechanisms. First, the 120 warning: the initial diagnosis and ECG carried out in the ambulance created first-hand data. The 120 warning was sent to the emergency triage system directly by telephone and started the green channel immediately. Second, the combination of the emergency clinical pathway, the emergency diagnosis system, and the green channel system, ensures that patients with STEMI do not have to wait. Third, ECG examinations were carried out sooner; patients with chest pain were guided by a nurse to complete ECG examinations as soon as possible so as to avoid a delay in treatment and promote the rapid progression of follow-up diagnosis and treatment. Fourth, the use of point of care testing (POCT) such as rapid TnI represents a valuable step. Finally, antiplatelet drugs are provided in one box so that they can be located quickly. The emergency clinical pathway includes different aspects of clinical and nursing work. Medical staff actively participate in the continuous quality improvement of the STEMI management process and jointly promote the realization of treatment objectives; this system is highly advantageous for the patients involved.

It is relatively common for patients with STEMI to experience serious complications. Some patients suffer from malignant arrhythmia and cardiogenic shock when they first arrive at the emergency room. Due to their critical condition, emergency physicians should commence the rescue process according to the emergency information system. The emergency physician should establish a vein pathway and protect the airway and circulation. PCI is now performed for vascular reperfusion by cardiologists under monitoring. The rapid availability of emergency physicians provides a guarantee for follow-up treatment. Efficient cooperation between departments is vital if we are to ensure the safety of patients.

Another factor that affects the goal time is the initiation of PCI. This requires that equipment and professionals can become available rapidly. The hospital manager should ensure that the arrangement of the PCI room is appropriate and that there is a specific emergency plan in place; this will allow the PCI team to respond rapidly. The hospital should formulate a series of management systems and provide training for relevant departments, especially with regards to professional skills in the emergency department. The hospital should also organize emergency drills on a regular basis. The realization of this part requires decision-making and persistent promotion at senior levels within the hospital. Information system records can provide effective data feedback to evaluate the efficacy of the improvement measures. The vitality of a clinical pathway is reflected in the continuous self-evaluation and renewal of clinical practice. The proportion of patients who were transferred to another hospital during the initial phases was high but improved over time.

There are some limitations to our information system that need to be considered. For example, the software is operated independently by emergency physicians independently; however, the clinical pathway for STEMI begins with getting the patients to hospital. An ideal information system should begin with pre-inspection, timing at every point, and information should be transferred by the unit at the next level. Each member of personnel completed the work within the period required by quality control requirements and submitted the reasons for any deviations from the clinical pathway. The final receiver was able to review all details of the process to formulate more individualized treatment plans for patients. This type of system will integrate all participants in diagnosis and treatment into one system to improve handover efficiency and reduce the errors caused by link changes, thus leading to a safer and more effective protocol.

## Conclusion

This study applied a new emergency clinical pathway to STEMI patients over a specific period of time and generated an information system for emergency situations according to a specific clinical pathway. The efficacy of the clinical pathway in flow management was evaluated over 6 years of data. The system is highly advantageous in that it considers each link in the clinical process for STEMI and continuously improves data quality.

## Data Availability Statement

The raw data supporting the conclusions of this article will be made available by the authors, without undue reservation.

## Author Contributions

ZX and JC analyzed and revised the manuscript. ZG and HZ collected the data. JC, KM, and ZX designed the study. All authors contributed to the article and approved the submitted version.

## Conflict of Interest

The authors declare that the research was conducted in the absence of any commercial or financial relationships that could be construed as a potential conflict of interest.

## Publisher’s Note

All claims expressed in this article are solely those of the authors and do not necessarily represent those of their affiliated organizations, or those of the publisher, the editors and the reviewers. Any product that may be evaluated in this article, or claim that may be made by its manufacturer, is not guaranteed or endorsed by the publisher.

## References

[1] Hagiwara MA Bremer A Claesson A Axelsson C Norberg G Herlitz J. The impact of direct admission to a catheterisation lab/CCU in patients with ST-elevation myocardial infarction on the delay to reperfusion and early risk of death: results of a systematic review including meta-analysis. *Scand J Trauma Resusc Emerg Med.* (2014) 22:67. 10.1186/s13049-014-0067-x 25420752 PMC4258278

[2] Scholz KH Maier SKG Maier LS Lengenfelder B Jacobshagen C Jung J Impact of treatment delay on mortality in ST-segment elevation myocardial infarction (STEMI) patients presenting with and without haemodynamic instability: results from the German prospective, multi-centre FITT-STEMI trial. *Eur Heart J.* (2018) 39:1065–74. 10.1093/eurheartj/ehy004 29452351 PMC6018916

[3] Greulich S Mayr A Gloekler S Seitz A Birkmeier S Schäufele T Time-dependent myocardial necrosis in patients with ST-segment–elevation myocardial infarction without angiographic collateral flow visualized by cardiac magnetic resonance imaging: results from the multi-center STEMI-SCAR project. *J Am Heart Assoc.* (2019) 8:e012429. 10.1161/JAHA.119.012429 31181983 PMC6645633

[4] Thygesen K Alpert JS Jaffe AS Chaitman BR Bax JJ Morrow DA Fourth universal definition of myocardial infarction(2018). *J Am Coll Cardiol.* (2018) 72:2231–64. 30153967 10.1016/j.jacc.2018.08.1038

[5] Thygesen K Alpert JS Jaffe AS Simoons ML Chaitman BR White HD Third universal definition of myocardial infarction. *J Am Coll Cardiol.* (2012) 60:1581–98. 22958960 10.1016/j.jacc.2012.08.001

[6] Chinese Medical Association, Chinese Medical Journals Publishing House, Chinese Society of General Practice, Editorial Board of Chinese Journal of General Practitioners of Chinese Medical Association, Expert Group of Guidelines for Primary Care of Cardiovascular Disease. Guideline for primary care of ST-segment elevation myocardial infarction (2019). *Chin J Gen Pract.* (2020) 19:1083–91.

[7] Chinese Society of Cardiology of Chinese Medical Association, Editorial Board of Chinese Journal of Cardiology. 2019 Chinese Society of Cardiology(CSC)guidelines for the diagnosis and management of patients with ST-segment elevation myocardial infarction. *Chin J Cardiol.* (2019) 47:766–83. 10.3760/cma.j.issn.0253-3758.2019.10.003 31648459

[8] Fraticelli L Kleitz O Claustre C Eydoux N Peiretti A Tazarourte K Comparison of the pathways of care and life courses between first-time ST-elevation myocardial infarction (STEMI) and STEMI with prior MI: findings from the OSCAR registry. *BMJ Open.* (2020) 10:e038773. 10.1136/bmjopen-2020-038773 33154054 PMC7646338

[9] Dhaliwal JS Goss F Whittington MD Bookman K Ho PM Zane R Reduced admission rates and resource utilization for chest pain patients using an electronic health record-embedded clinical pathway in the emergency department. *J Am Coll Emerg Physicians Open.* (2020) 1:1602–13. 10.1002/emp2.12308 33392569 PMC7771814

[10] Ferry AV Strachan FE Stewart SD Marshall L Lee KK Anand A Exploring patient experience of chest pain before and after implementation of an early rule-out pathway for myocardial infarction: a qualitative study. *Ann Emerg Med.* (2020) 75:502–13. 10.1016/j.annemergmed.2019.11.012 31983496 PMC7105816

[11] Kinsman LD Buykx P Humphreys JS Snow PC Willis J. A cluster randomised trial to assess the impact of clinical pathways on AMI management in rural Australian emergency departments. *BMC Health Serv Res.* (2009) 25:83. 10.1186/1472-6963-9-83 19463196 PMC2688500

[12] Stepinska J Lettino M Ahrens I Bueno H Garcia-Castrillo L Khoury A Diagnosis and risk stratification of chest pain patients in the emergency department: focus on acute coronary syndromes. A position paper of the Acute Cardiovascular Care Association. *Eur Heart J Acute Cardiovasc Care.* (2020) 9:76–89. 10.1177/2048872619885346 31958018

[13] Khatib R Patel N Laverty U Mcgawley G McLenachan J Shield S Re-engineering the post-myocardial infarction medicines optimisation pathway: a retrospective analysis of a joint consultant pharmacist and cardiologist clinic model. *Open Heart.* (2018) 5:e000921. 10.1136/openhrt-2018-000921 30613417 PMC6307610

[14] Than MP Pickering JW Aldous SJ Cullen L Frampton CM Peacock WF Effectiveness of EDACS versus ADAPT accelerated diagnostic pathways for chest pain: a pragmatic randomized controlled trial embedded within practice. *Ann Emerg Med.* (2016) 68:93–102. 10.1016/j.annemergmed.2016.01.001 26947800

[15] Riley RF Miller CD Russell GB Harper EN Hiestand BC Hoekstra JW. Cost analysis of the history, ECG, age, risk factors, and initial troponin (HEART) pathway randomized control trial. *Am J Emerg Med.* (2017) 35:77–81. 10.1016/j.ajem.2016.10.005 27765481 PMC5189662

[16] Starmer G Schrale R. Heart of the tropics: delivering evidence-based care for acute coronary syndromes in northern Australia. *Rural Remote Health.* (2016) 16:3938. 27817198

[17] Park YS Chung SP You JS Kim MJ Chung HS Hong JH. Effectiveness of a multidisciplinary critical pathway based on a computerised physician order entry system for ST-segment elevation myocardial infarction management in the emergency department: a retrospective observational study. *BMJ Open.* (2016) 6:e011429. 10.1136/bmjopen-2016-011429 27531726 PMC5013344

[18] Hofer TP Hayward RA. Are bad outcomes from questionable clinical decisions preventable medical errors? A case of cascade iatrogenesis. *Ann Intern Med.* (2002) 137(5 Pt 1):327–33. 10.7326/0003-4819-137-5_part_1-200209030-00008 12204016

[19] Chapman AR Anand A Boeddinghaus J Ferry AV Sandeman D Adamson PD. Comparison of the efficacy and safety of early rule-out pathways for acute myocardial infarction. *Circulation.* (2017) 135:1586–96. 10.1161/CIRCULATIONAHA.116.025021 28034899 PMC5404406

[20] McDermott KA Helfrich CD Sales AE Rumsfeld JS Ho PM Fihn SD. A review of interventions and system changes to improve time to reperfusion for ST-segment elevation myocardial infarction. *J Gen Intern Med.* (2008) 23:1246–56. 10.1007/s11606-008-0563-7 18459014 PMC2517976

[21] Habib H Ginanjar E Mansjoer A Sulistio S Albar IA Mulyana RM. ST-Elevation myocardial Infarction: a simulation case for evaluation of interprofessional performance in a hospital. *Emerg Med Int.* (2019) 2019:7562637. 10.1155/2019/7562637 31687214 PMC6800974

[22] Dunn F Hughes D Rocke L McNicholl B. Are chest pain observation units essential for rapid and effective emergency care in the UK? *Emerg Med J.* (2006) 23:487–8. 10.1136/emj.2005.023671 16714524 PMC2564360

[23] Rafi A Sayeed Z Sultana P Aik S Hossain G. Pre-hospital delay in patients with myocardial infarction: an observational study in a tertiary care hospital of northern Bangladesh. *BMC Health Serv Res.* (2020) 20:633. 10.1186/s12913-020-05505-x 32646521 PMC7346615

[24] Dijkema LM Dieperink W van Meurs M Zijlstra JG. Preventable mortality evaluation in the ICU. *Crit Care.* (2012) 16:309. 10.1186/cc11212 22546292 PMC3681346

[25] Wang L Zhang M Guo L Qi J Luo H He H Clinical pathways based on integrative medicine in Chinese hospitals improve treatment outcomes for patients with acute myocardial infarction: a multicentre, nonrandomized historically controlled trial. *Evid Based Complement Alternat Med.* (2012) 2012:821641. 10.1155/2012/821641 23024695 PMC3450432

[26] Cheung GS Tsui KL Lau CC Chan HL Chau CH Wu KL Primary percutaneous coronary intervention for ST elevation myocardial infarction: performance with focus on timeliness of treatment. *Hong Kong Med J.* (2010) 16:347–53. 20889998

[27] Mahler SA Riley RF Russell GB Hiestand BC Hoekstra JW Lefebvre CW Adherence to an accelerated diagnostic protocol for chest pain: secondary analysis of the HEART pathway randomized trial. *Acad Emerg Med.* (2016) 23:70–7. 10.1111/acem.12835 26720295 PMC4716613

